# Unlocking latent kinetic information from label-free binding

**DOI:** 10.1038/s41598-019-54485-4

**Published:** 2019-12-05

**Authors:** John G. Quinn, Micah Steffek, John M. Bruning, Alexandra Frommlet, Melinda M. Mulvihill

**Affiliations:** Biophysical group, Biochemical and Cellular Pharmacology, Genentech, Inc., 1 DNA Way, South San Francisco, CA 94080 USA

**Keywords:** Biophysics, Molecular biophysics

## Abstract

Transient affinity binding interactions are central to life, composing the fundamental elements of biological networks including cell signaling, cell metabolism and gene regulation. Assigning a defined reaction mechanism to affinity binding interactions is critical to our understanding of the associated structure-function relationship, a cornerstone of biophysical characterization. Transient kinetics are currently measured using low throughput methods such as nuclear magnetic resonance, or stop-flow spectrometry-based techniques, which are not practical in many settings. In contrast, label-free biosensors measure reaction kinetics through direct binding, and with higher throughout, impacting life sciences with thousands of publications each year. Here we have developed a methodology enabling label-free biosensors to measure transient kinetic interactions towards providing a higher throughput approach suitable for mechanistic understanding of these processes. The methodology relies on hydrodynamic dispersion modeling of a smooth analyte gradient under conditions that maintain the quasi-steady-state boundary layer assumption. A transient peptide-protein interaction of relevance to drug discovery was analyzed thermodynamically using transition state theory and numerical simulations validated the approach over a wide range of operating conditions. The data establishes the technical feasibility of this approach to transient kinetic analyses supporting further development towards higher throughput applications in life science.

## Introduction

Affinity interactions occur over an extraordinarily wide range in kinetics in order to support a diversity of life-sustaining processes. Understanding the associated structure-function relationship^1^ requires assigning a defined reaction mechanism^2^, which may be accomplished through kinetic analysis. Transient protein-protein interactions are ubiquitous in cell biology^3^ and almost all enzymes interact transiently with substrates^4^, or each other^5^, in order to promote high catalytic turnover. Indeed the majority of early chemical matter discovered in small molecule drug discovery are transient binding compounds. Generally, kinetic interaction constants are not predictable^6^ from knowledge of structure due to their complexity^1,6,7^ but are routinely measured using biophysical techniques^8^. Transient kinetics are most often measured by lower throughput methods such as NMR^9^, or stop-flow spectrometry-based techniques^10–12^, thereby restricting their use and limiting our understanding and exploitation of transient binding interactions. Label-free optical biosensing has become the standard for measuring direct binding of slow-to-moderate kinetics allowing mechanistic analysis^13–16^ at reasonably high throughput. Indeed the approximate number of publications mentioning surface plasmon resonance (SPR), which is the most common detection mode, now approaches 70,000^17^. Therefore, the use of label-free biosensors to measure transient kinetic interactions holds high potential in many fields especially drug discovery. Briefly, conventional kinetic analysis of drugs reveals that an optimal therapeutic profile may depend on achieving a particular target-specific kinetic profile^16,18,19^. This suggests that kinetic and mechanistic profiling should be exploited throughout early drug discovery in order to provide more optimal starting points for lead development by favoring a given kinetic “sweet spot”. At a minimum, we envision that mechanistic characterization of transiently bound early hits through kinetic profiling may prove more effective in discriminating artifactual binding^20–22^ from tractable binding modes.

We have developed an approach that adapts real-time flow-injection-based biosensors to measure transient kinetics. Briefly, microfluidic geometries employed in current flow injection analysis (FIA)-based systems are relatively large (effective hydrodynamic diameter ≥ 50 μm) in order to maintain robust low pressure sampling and instrument developers^23^ have focused on minimizing microchannel “non-swept dead volumes” in order to rapidly establish a uniform analyte concentration upon injection. Despite these efforts injection rise/fall regions remain >200 ms thereby limiting the uniform concentration approximation to kinetics >500 ms. We postulated that the kinetic limit of detection could be extended by including a hydrodynamic analyte dispersion term to model changing analyte concentrations within injection rise/fall regions. We evaluate this approach using test data generated by multiphysical numerical simulation and we report an experimental proof-of-principle relevant to drug discovery. We define moderate transient kinetics to be in the order of 50–500 ms and fast transient kinetics to be in the order of 1–50 ms. For a simple 1:1 binding interaction the equilibrium dissociation affinity constant is *K*_*D*_ = *k*_*d*_*/k*_*a*_ (units, M), where *k*_*a*_ (units, M^−1^ s^−1^) is the association rate constant, *k*_*d*_ (units, s^−1^) is the dissociation rate constant and *τ* = *1/k*_*d*_ (units, s) is the residence time. The equilibrium response is *R*_*eq*_ = *R*_*max*_*c/*(*c* + *K*_*D*_), where *R*_*max*_ (unit, RU) is the saturation response and *c* is the analyte concentration (units, M). We employed unmodified commercially available technology for experimental work but instrument modifications will be required for optimal implementation including, parallel sensing spots that are short in the flow direction, optimized dispersion profiles and higher time resolution.

## Results

### Injection-binding process

**Figure 1:**
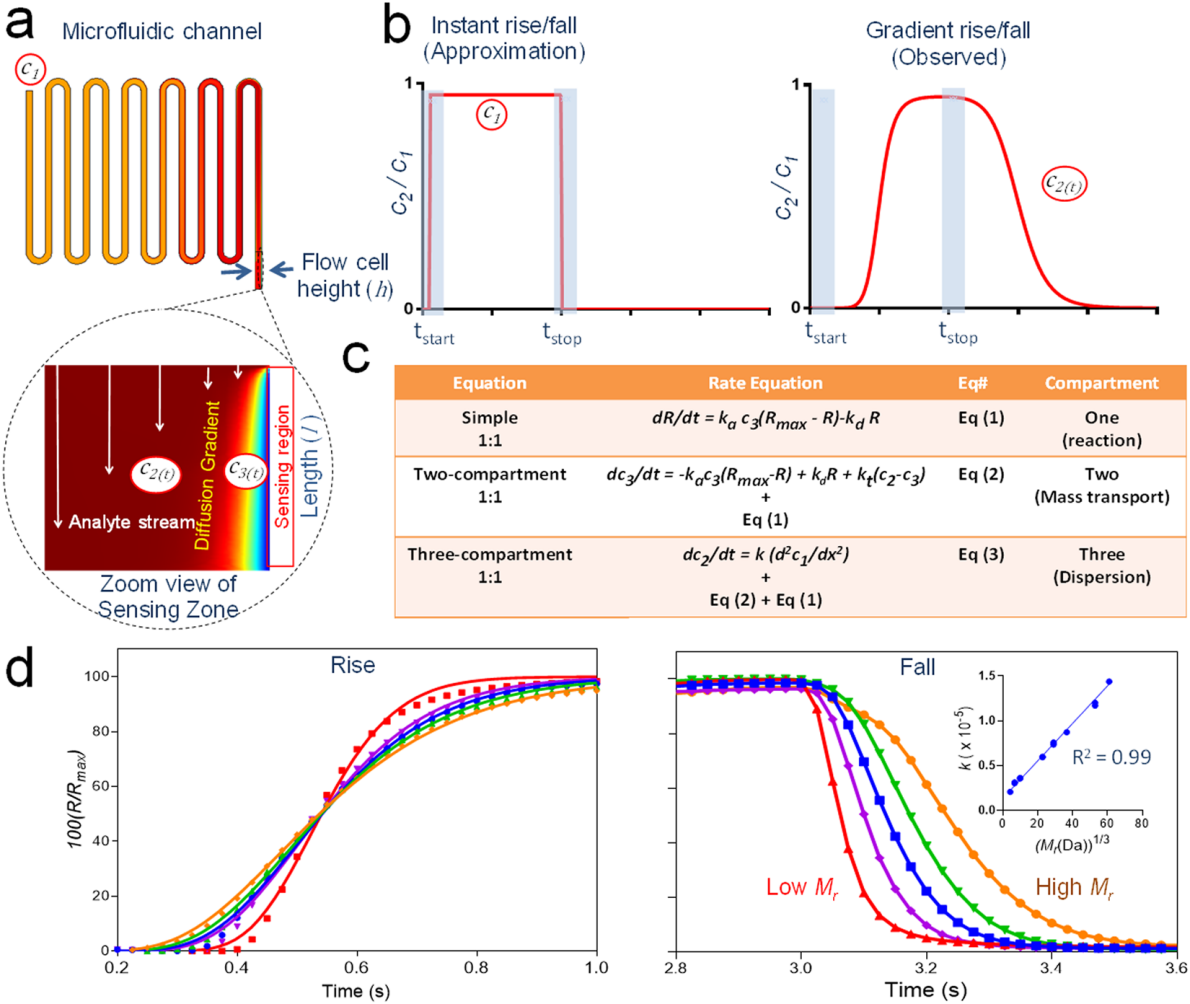
Compartment models in label-free biosensing. (**a**) Numerical simulation of a full injection-binding process with numerically computed analyte concentration gradients within the microchannel (length, *l* and height, *h*, where *x* is the distance along the channel length) and associated flow cell rendered as a color gradient. Concentrations increase from blue-to-red and the parabolic velocity field within the flow cell is depicted by vertical arrows with zero flow at the walls. (**b**) Simulated analyte concentration profiles (red) for instant rise/fall injection of a uniform concentration and a more realistic gradient rise/fall. Turbulence upon injector actuation is indicated by grey panels. (**c**) 1:1 binding interaction models. The associated model parameters are defined in the introduction. (**d**) Experimentally measured bulk RI-based dispersion curves for compounds ranging in *M*_*r*_ from 342 Da to 670 kDa. Equation S1 (see supplemental information) is a dispersion equation and was fit locally (average *R*^2^ = 0.998) to the fall dispersion points and a subset of five curves is shown. The inset panel shows *k* as a function of *M*_*r*_^1/3^ with a linear fit given by *k* = 2.09 *×* 10^−*7*^ *M*_*r*_^*1/3*^ + 1.34 *×* 10^−*6*^). Here all eight *M*_*r*_ standards were included in duplicate.

The time evolution of analyte concentration through the injection-binding process is shown in Fig. 1a and includes three compartments as described by Quinn^24,25^ previously. In previous work a coiled capillary provided a third compartment for the generation of slowly evolving (i.e. >30 s) analyte gradients. Here we have not added additional capillaries/microchannels and instead demonstrate that rise/fall regions associated with washout of residual sub-μL dead volumes generate sub-second dispersion gradients that are well suited to the analysis of transient kinetics. By convention these gradient regions are discarded but here we extend the dynamic range of optical label-free biosensing by incorporating these rise/fall regions using a three-compartment model that now allows accurate kinetic measurements into the millisecond range. Briefly, analyte at a fixed concentration *c*_1_, is injected into a microfluidic channel and disperses into pre-existing buffer (compartment 3), because of both convective and diffusive mixing, and is quantified by a dispersion coefficient *k*. The analyte dispersion arrives at the flow cell as a time-dependent concentration gradient *c*_2(t),_ where a fraction of the analyte diffuses through a stagnant boundary layer (compartment 2) to reach the target-coated sensing surface (compartment 1) at concentration c_3(t)_. There the binding reaction of analyte with bound target is measured in real-time through label-free optical detection. Figure 1b shows the analyte concentration profile for an instant rise/fall injection (left) with a uniform concentration assumed throughout the injection. However, in reality the injected sample will disperse into a non-negligible dead-volume to produce a gradient in analyte concentration at the rise/fall regions of injections. Turbulence exists briefly upon injector actuation due to transient pressure/temperature fluctuations and the dead-volume upstream of the flow cell causes a time offset with respect to analyte arrival at the flow cell thereby allowing re-establishment of laminar flow, a pre-requisite to reproducible kinetic measurements.

A simple 1:1 Langmuir model (Fig. 1c) is given by equation (1) and assumes that analyte gradients do not develop. A 1:1 two-compartment model couples equations (1) and (2) and assumes that a gradient in analyte concentration can exist at the surface. This gradient results in mass transport limitation (MTL) defined by a single mass transport rate constant *k*_*t*,_ which limits the flux of analyte through the boundary. Similarly, the dispersion gradient has a significant impact at short injection times requiring a three-compartment model that couples equations (1) and (2) with a microchannel dispersion term, as given by equation (3). In order to experimentally assess the quality of analyte dispersions, a series of *M*_*r*_ standards were injected and the resulting bulk refractive index (RI) dispersions (Fig. 1d) showed relatively weak *M*_*r*_-dependence in the rise-dispersion, indicating convection-limited dispersion, and the fall dispersions returned dispersion constants (*k*) that were proportional to *M*_*r*_^1/3^, indicating diffusion-limited dispersion.

### Low MTL/Moderate transient regime

**Figure 2:**
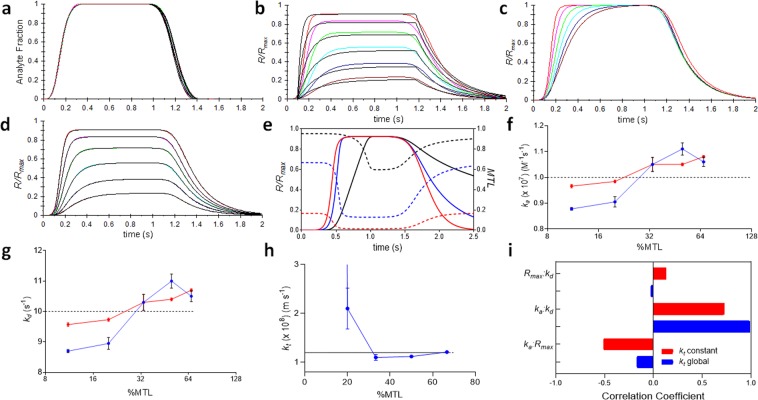
Validation of the three-compartment model using numerically simulated binding curves. The interaction constants for all simulated binding curves are given in table S1 (Supplemental information). (**a**) Overlay of analyte dispersion profiles (i.e. color) for six serial-doubling dilution injections fit to equation S2 (supplemental information). (**b**) Affinity binding curves (color) for the analyte injections in (**a**) binding to surface-bound target and fit to a two-compartment model (black), where *R*_*max*_ was 1.5 RU. (**c**) Binding curves in (**b**) normalized with respect to each respective equilibrium response, *R*_*eq*_. (**d**) Affinity binding curves in (**b**) fit to a three-compartment model. (**e**) Affinity binding curves for the interaction of analyte at *R*_*max*_ settings of 0.5 RU (red), 5 RU (blue) and 50 RU (black). %MTL decreases as surface binding progresses as indicated by the broken lines color matched to each respective *R*_*max*_ condition. (**f**,**g**,**h**) Interaction parameters returned from a fit to a three-compartment model as a function of %MTL, for *k*_*a*_ (**f**), k_d_ (**g**) and *k*_*t*_ (**h**), respectively, where *k*_*t*_ was pre-calculated (red) from equation S3 (supplemental information), or fit globally (blue). The dotted line is the “true” parameter value and the error bars are ± SE. All χ^2^ values were < 0.08%. (**i**) Parameter correlation analysis for three-compartment model (Refer to supplemental information for more details).

Transient binding response curves (Fig. 2a–d) were simulated for *k*_*a*_ = 1 × 10^6^ M^−1^s^−1^, *k*_*d*_ = 5 s^−1^ and *R*_*max*_ = 1.5 RU (equivalent to 3 pmoles mm^−2^) producing <1.5% MTL. Typical sample contact times are >10 s but here we have reduced this time to just 1 s, a more suitable time scale for analysis of moderate transient kinetics. The time-dependent analyte concentration profiles for each injected concentration (Fig. 2a) were fit to equation (S2) (supplemental information) producing superimposable fits where the residual difference was < 1%. The associated binding curve set (Fig. 2b) was fit to a two-compartment model producing high systematic deviation (>40%). Each curve was normalized with respect its maximum response causing the curve set to superimpose (Fig. 2c) other than during the association phase. A three-compartment model was fit (Fig. 2d) returning kinetic constants with <1% error relative to “true” values and <0.05% SE associated with each parameter. The average squared residual χ^2^ was 7.4 × 10^−4^ RU and the maximum systematic residual deviation was <2%.

### Variable MTL/ moderate transient regime

A time-dependent MTL factor was plotted for three simulated dispersion curves, for a serial ten-fold increase in *R*_*max*_ at a fixed analyte concentration, and is shown along with each respective binding curve in Fig. 2e. The simulated curve set in Fig. 1b was replicated at five *R*_*max*_ values corresponding to a range of 11–66%MTL. A three-compartment model was fit to each curve set and the parameter values returned from the fit were plotted with respect to %MTL. Figure 2f–h. Pre-calculating *k*_*t*_ reduced error in kinetic parameters to <5% when ≤ 50%MTL. The error associated with global fitting of *k*_*t*_ decreased with increasing %MTL and increased exponentially at <20% MTL becoming undefined at 11%MTL. The parameter correlation analysis in Fig. 2i was performed at 50% MTL and shows that kinetic constants were highly correlated and this was reduced significantly when *k*_*t*_ was pre-calculated.

### Experimental characterization in a moderate transient regime

**Figure 3:**
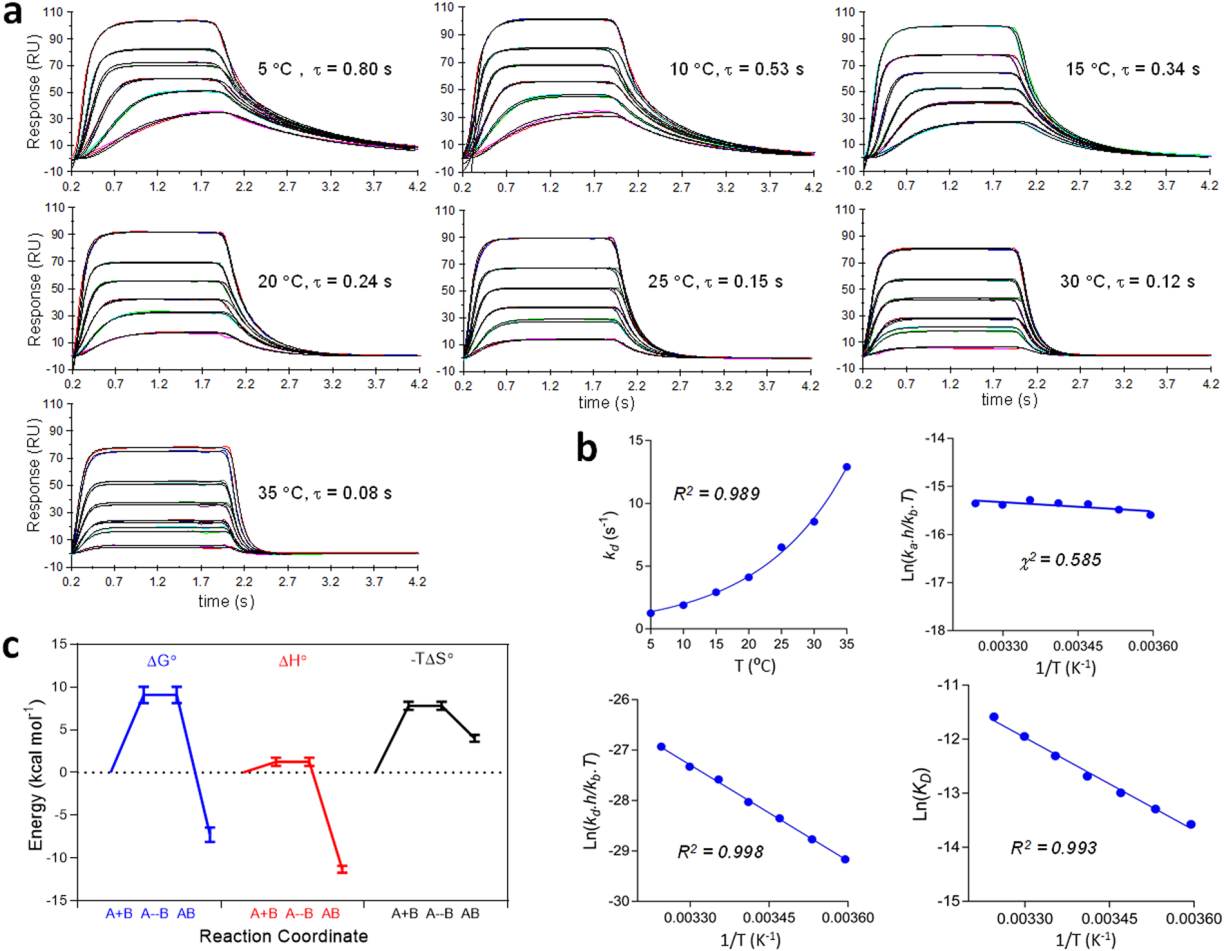
Experimentally measured temperature-dependence of peptide binding to MBP-tagged target molecule using analyte gradient injections. (**a**) Three-compartment model fit containing a bulk RI term and fit to each binding curve set with *k*_*a*_, *k*_*d*_ and *R*_*max*_ fit as global parameters. *k*_*t*_ was pre-calculated from equation S3 (supplemental information) at each temperature and fit as a constant. Each curve set contains six curves produced from injection of a peptide over a serial doubling dilution range replicated in duplicate. High reproducibility and goodness of fit cause both the replicate curves (color) and the fitted model curves (black) to appear superimposable. (**b**) Thermodynamic models were fit to the data generating a thermodynamic profile. (Top left) Exponential increase in *k*_*d*_ with temperature *k*_*d*_ = 0.94^(0.*0747/T*)^. (Bottom left) Linear Erying plot for *k*_*d*_. (Top right) Linear Erying plot for *k*_*a*_. (Botom right) Linear Van’t Hoff plot. (**c**) Energy transitions at 25 °C as a function of the reaction coordinate for the thermodynamic parameters obtained from (**b**).

Binding of a ~3.8 kDa peptide to a maltose binding protein (MBP)-tagged target was evaluated over a dose-response range at seven temperatures from 5–35 °C and the fitted binding curve sets (Fig. 3a). The average SE associated with *k*_*a*_ and *k*_*d*_ was 1.7% and 0.80%, respectively, and the average χ^2^ over all data sets was 0.75%. The interaction became transient over the temperature range with τ decreasing ~10-fold to τ ≈ 0.08 s, and is also plotted in terms of *k*_*d*_ in Fig. 3b. *k*_*a*_ was relatively insensitive to temperature, increasing by ~1.4-fold from 1.0 × 10^6^ M^−1^ s^−1^ over the temperature range. Greater random variation was observed in *k*_*a*_ estimates relative to *k*_*d*_ and may have been related to convection dominated dispersion during the rise dispersion (Fig. 1d) and possibly greater variation in pressure-induced microchannel compliance. Indeed, *k*_*a*_ was more reliably estimated from *k*_*a*_ = *k*_*d*_*/K*_*D*_ which was employed for Erying analysis. The Erying plots (Fig. 3b) for both kinetic constants were linear, as was the Van’t Hoff analysis of the *K*_*D*_, and the resulting thermodynamic parameters are shown in Fig. 3c as energy transitions with respect to the reaction coordinate.

### Fast transient regime

**Figure 4:**
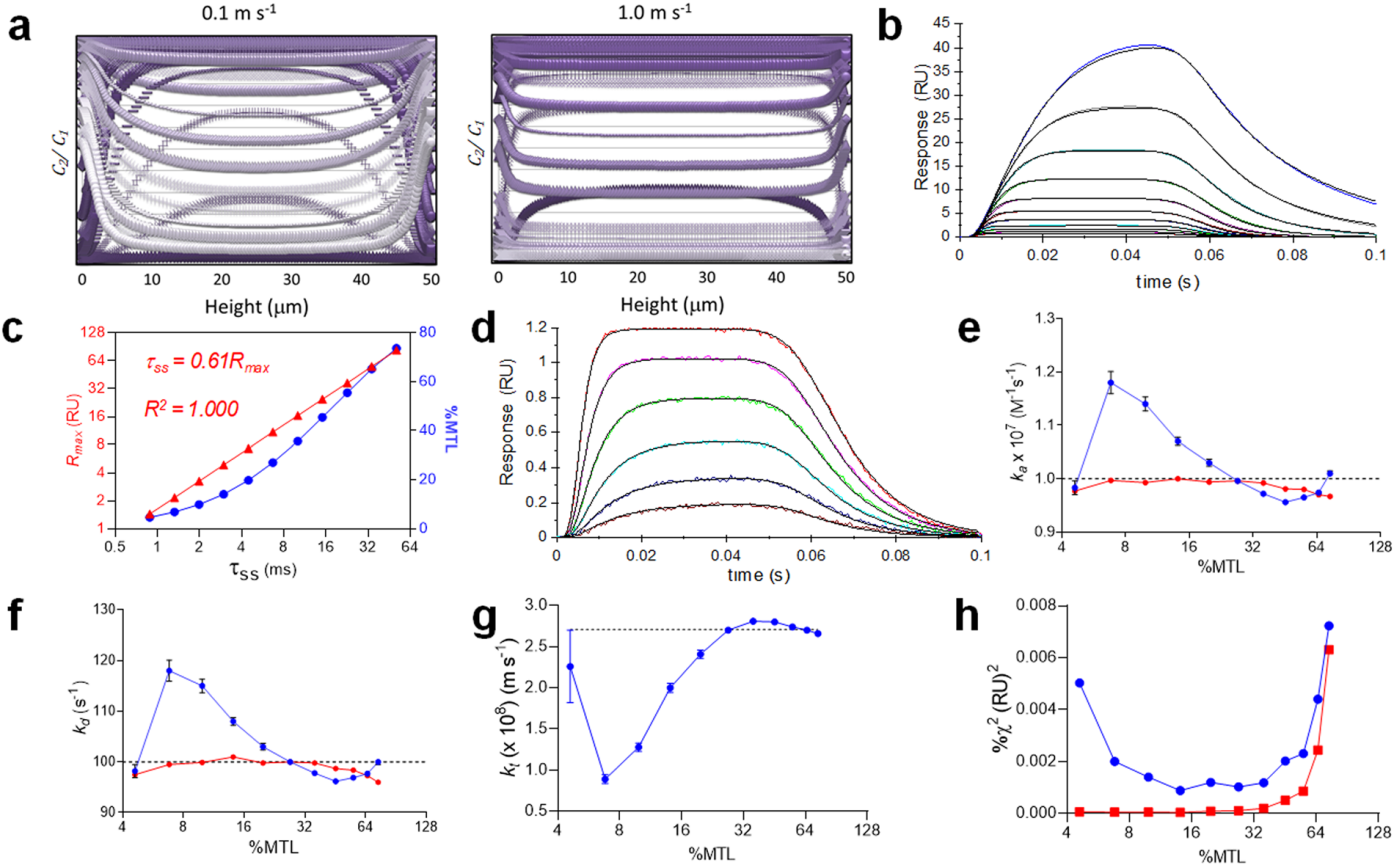
Simulation study of boundary layer formation and its effect on estimation of fast transient kinetics. (**a**) Superimposed analyte concentration profiles across the height of the flow cell at 2 ms intervals over the course of a single analyte injection. (**d**) Boundary layer curves (color) at a fixed concentration *c*_1_ = *K*_*D*_ over a serial-doubling range in *R*_*max*_, where reaction kinetics were made semi-infinite allowing *k*_*t*_ to drive occupancy. A three-compartment model was fit (superimposed black curves) returning *k*_*t*_, where *R*_*max*_ and *K*_*D*_ were held constant. **C** *R*_*max*_ and %MTL as a function of *τ*_*ss*_ for the binding curves in (**b**). (**d**) Three-compartment model fit (superimposed black curves) to numerically simulated curves (color) with low *R*_*max*_ and with pseudo-random noise added as a normal distribution where root mean square standard deviation ≈ 0.015 RU, which is typical of current state-of-the art systems. The curve set was simulated over a serial-doubling dose response range, where *R*_*max*_ ≈ 1.44 RU. (**e**,**f**,**g**) Interaction parameters returned from fitting a three-compartment model to the curve set in (**d**) replicated over a range in R_max_ producing a wide %MTL range, for *k*_*a*_ (**e**), k_d_ (**f**) and *k*_*t*_ (**g**), respectively. *k*_*t*_ was pre-calculated (red) from equation S3 (see supplemental information), or fit globally (blue). The dotted lines represent the “true” parameter value from the numerical simulation and the error bars are ± SE associated with fitting the parameter. **h**. χ^2^ values for the curve fits in (**e**), (**f**) and (**g**).

A numerical simulation of the analyte concentration across the height of the flow cell is shown at 2 ms intervals for an analyte dispersion injection in Fig. 4a. The flow cell can generally be taken as the region of the microchannel surrounding the sensing region along with all other target-coated regions upstream of the sensing region. The concentration rises to a maximum *c*_1_ and returns to zero when the gradient falls, where the fall concentration profiles are inverted (∪) relative to the preceding rising gradient profiles (∩). At a flow rate of 0.1 m s^−1^ the approximate washout time *t*_*wash*_ ≈ 3 ms (assuming *t*_*wash*_ ≈ *3t*_*tr*_, where *t*_*tr*_ is the time to traverse the flow cell) and a parabolic gradient profile was observed in the bulk flow with thin diffusion boundary layers at each wall.

### Boundary layer quasi-steady state approximation (BLQSSA)

We numerically simulated kinetic binding curves with pseudo-infinite kinetics (i.e. *k*_*a*_ = 10^10^ m^−1^s^−1^, *k*_*d*_ = 10^4^ s^−1^, *τ* = 0.1 ms) allowing boundary layer mass transport to dictate the analyte binding rate. Under these conditions the observed curvature became a function of *R*_*max*_, *R*_*eq*_ and *k*_*t*_ and a three-compartment model fit is shown in Fig. 4b. A quasi steady-state time, in this case associated with the development of the transport limited boundary layer, was approximated by τ_ss_ ≈ 1.5τ_t_, with a boundary decay time approximated as *τ*_*t*_ ≈ *R*_*max*_*/k*_t_*R_*eq*_. *V*ariation in both *R*_*max*_ and %MTL with respect to τ_ss_ is shown in Fig. 4c. The influence of high MTL on transient kinetics was evaluated by fitting a three-compartment model to curves generated by numerical simulations for a fast transient interaction where *k*_*a*_ = 1 × 10^7^ M^−1^s^−1^ and *k*_*d*_ = 100 s^−1^ where pseudo-random Gaussian noise typical of current instrumentation was added, as shown in Fig. 4d. %MTL was minimized by confining the surface bound target to small sensing regions specifically avoiding coating upstream of the sensing regions. These simulations were repeated over a range in *R*_*max*_ producing curve set exhibiting low-to-high %MTL. Kinetic constants returned from model fitting are shown (Fig. 4 e,f), where *k*_*t*_ was fit either globally, or held constant at a value calculated from equation (S3). The error in both *k*_*a*_ and *k*_*d*_ was ≤ 4% when *k*_*t*_ was held constant and χ^2^ values were reduced significantly (Fig. 4h) supporting this approach. Indeed *k*_*t*_ values returned from global fitting (Fig. 4g) were highly uncertain (~250% error) when %MTL was low, again implying that low MTL destabilizes the fit as indicated by higher χ^2^ values (Fig. 4h).

## Discussion

The development and application of computational models of label-free biosensing is well understood with several insightful publications^26,27^. A full numerical model is sophisticated and not suitable for fitting experimental data for estimation of reaction kinetics. It does however generate binding curves defined by absolute kinetic values, while retaining the complexities of experimental data, thereby providing an absolute standard for quantitative testing of simpler mechanistic models. The time evolution of the injection-binding process was determined by multiphysical simulation as shown in Fig. 1a. Conventionally a uniform concentration profile (Fig. 1b) can be assumed to reliably approximate experimental binding curves but this assumption does not hold for rapid injections where rise/fall zones containing analyte gradients constitute a significant fraction of the injection time. When SPR-based biosensors first became available over three decades ago, a simple 1:1 Langmuir model (equation (1) in Fig. 1c), which assumes that analyte gradients do not develop, was thought sufficient to model simple binding interactions. However, it was soon realized that the analyte flux to the sensing surface can become limiting and a 1:1 two-compartment model (coupled equations (1) and (2) in Fig. 1c) has since become the gold standard and was validated by computational modeling^28^. In the two-compartment model the analyte concentration at the surface was approximated by adding a mass transport reaction defined by a single mass transport rate constant that drives a reversible flux of analyte through the diffusion boundary layer to the sensing surface. When the binding reaction flux coefficient *k*_*r*_ = *k*_*a*_ (*R*_*max*_*-R*) at the sensing surface is in the same order, or higher, than the mass transport coefficient *k*_*t*_, then significant mass transport limitation *MTL* = *k*_*r*_*/*(*k*_*r*_ + *k*_*t*_) will occur causing an analyte gradient to form. Another unrelated analyte gradient develops through dispersive mixing within microchannel(s) en-route to the flow cell but is invariably neglected because it is assumed fast relative to reaction kinetics. This assumption does not hold for transient reaction kinetics and a gradient dispersion term must be included as a three-compartment model, by coupling equations (1), (2) and (3) (see Fig. 1c), providing a more realistic model of the injection-binding process. These analyte dispersions (Fig. 1d) were highly reproducible and evolved in a sub-second time regime suitable for the study of transient reaction kinetics. A set of numerically generated dispersion curves (Fig. 2a) were found to fit well to equation (S2) (supplemental information) and this equation was therefore selected for all kinetic modeling.

In contrast to standard uniform injections, both dispersion and binding kinetics contribute to observed curvature of binding response curves in a transient time regime. Briefly, for a simple 1:1 interaction, an idealized uniform analyte injection should produce monotonic binding curvature towards surface saturation, as implied by the observed reaction rate *k*_*obs*_ = *k*_*a*_*c* + *k*_*d*_, where *c* = constant. However, dispersion defines time-dependent changes in analyte concentration that induce additional concentration-dependent curvature that becomes apparent by fitting a two-compartment model, which cannot account for dispersion and therefore shows high systematic deviation (Fig. 2b). More importantly, inappropriate model fitting can easily occur when τ < data acquisition rate because all response points are at quasi-steady-state^25^ and any observed curvature must be derived from dispersion alone, yet can easily be mistaken as kinetic curvature. However, by normalizing each curve with respect to *R*_*max*_, curves without kinetic curvature superimpose and curves possessing kinetic curvature exhibit non-superimposable dose-dependent curvature in the association phase (Fig. 2c). The three-compartment model accounts for dispersion and producing an ideal fit (Fig. 2d) that returned kinetic constants with <1% error.

MTL scales with the number of free target sites (*R*_*max*_*-R*), which decrease as occupancy increases, allowing kinetic curvature to exist on approach to saturation (Fig. 2e) even at very high MTL conditions. In practice, highly MTL binding curves are often recorded before assay conditions have been optimized for a given panel of interactants and confining the fitted model to these”kinetic regions” can provide an approximate kinetic analysis. MTL delays both association, and dissociation, phases and therefore increases parameter correlation yet does not greatly impact parameter return at moderate level^28^. In order to demonstrate that this holds for transient kinetics, we generated multiple simulated curve sets over a wide range of MTL conditions and the parameter values returned from fitting the three-compartment model were determined (Fig. 2f,g). We found that *k*_*t*_ estimates from equation (S3) was 1.20 × 10^8^ ms^−1^, which was in good agreement with the value returned from a global fit (i.e. 1.21 × 10^8^ m s^−1^) at 67% MTL. While the three-compartment model performed well for all MTL levels tested, the error in kinetic constants was at a minimum at ~30% MTL, suggesting that pre-calculation of *k*_*t*_^29^ may increase fit stability. In the case of the standard two-compartment model it has been shown that kinetic constants are highly correlated when *k*_*t*_ is fit as a global parameter yet the high information content of binding response curves ensures accurate parameter return. Similarly, high parameter correlations were observed for the three-compartment model (Fig. 2i) at 50% MTL but these decreased significantly upon pre-calculation of *k*_*t*_ improving the stability of the fitted model.

In contrast to numerical simulations, kinetic binding constants for actual bimolecular interactions are not absolute quantities thereby complicating model validation through experiment. However, kinetic binding becomes more transient with increasing temperature, providing a range of kinetic constants for a given compound that are related through a common thermodynamic relationship. This relationship can be exploited to validate our model since inaccurate transient kinetic constants would be identified as outliers from the fitted thermodynamic profile. For this analysis (Fig. 3) we employed a peptide-protein interaction exhibiting kinetic constants approaching the current kinetic limit of detection at low temperatures and unmeasurable transient kinetics at high temperatures. Binding was evaluated from 5–35 °C and each binding curve set was fit to a three-compartment model (Fig. 3a) allowing thermodynamic analysis of the resulting interaction constants (Fig. 3b,c). The absence of outliers implied that transient kinetics was well determined from application of the three-compartment model and associated methods. This transient interaction has favorable enthalpy and there is a large entropy cost associated with activation of the transition state. Such mechanistic information may be exploited to validate and/or prioritize transiently bound chemical matter in early drug discovery and this first proof-of-principle experimentally demonstrated a 6-fold extension of kinetics into the transient regime.

Kinetic analysis of fast transient binding curves depends on high resolution injection clocking and data acquisition. Briefly, the time required to attain steady-state defines the relevant time regime required to measure transient kinetic constants and, by solving equation (1), assuming a fixed concentration, the time to attain a given steady-state occupancy is *t*_*Θ*_ = *-Ln* (1*-Θ*)*/*(*k*_*a*_*c* + *k*_*d*_), where *Θ* is fractional progress towards steady-state. Therefore at a concentration *c*_1_ = *K*_*D*_, the 95% steady-state time Θ = 0.95 will be approximately 1.5τ. This defines a threshold time resolution for the appearance of kinetic curvature and implies that a higher time resolution (e.g. ≤ 0.5τ) is needed for resolving kinetics. The maximum data acquisition rate available in currently available systems (i.e. ≤ 40 Hz) limits the measurement of transient kinetics and >10-fold increase may be required to measure transient binding kinetics routinely in drug discovery applications. Analyte concentration gradients in the bulk flow along the length, or height, of the flow cell must be minimal in order for mechanistic binding interaction models to hold. In practice this requires that the time delay associated with sample traversing the sensing regions *t*_*tr*_ ≪ τ. Numerical simulations (Fig. 4a) show that at a low flow rate a parabolic analyte concentration profile across the height of the flow flow cell is produced due to the influence of flow cell washout. At higher flow rates, washout becomes rapid relative to the evolution of the analyte gradient allowing a quasi-steady state concentration across the height of the flow cell.

With the improved sensitivity of current biosensors, it has become practical to employ a low *R*_*max*_ (1–5 RU) such that BLQSSA will hold under most practical experimental conditions. However, higher *R*_*max*_ values are often unavoidable in order to offset other sources of interference and this produces thicker boundary layers that take longer to form. This allows significant analyte binding before the boundary layer equilibrates, which violates BLQSSA. We numerically simulated the formation of the boundary layer over a range in *R*_*max*_, assuming pseudo-infinite reaction kinetic constants establishing approximately 100% MTL, thereby allowing pre-steady-state development of the boundary layer to be studied using the three-compartment model (Fig. 4b). The steady-state time associated with the development of the boundary layer was approximated by *τ*_*ss*_ *≈ 1*.5*τ*_*t*_, and the observed boundary decay time was found to be well approximated by *τ*_*t*_ ≈ *R*_*max*_*/k*_t_*R_*eq*_. *τ*_*ss*_ increased linearly with increasing *R*_*max*_ (Fig. 4c), extending into the moderate transient kinetic regime (>50 ms). For transient kinetics we might expect highest interference when *τ ≤ τ*_*ss*_ and to test this we numerically simulated experimentally realistic binding curves over a range in *R*_*max*_ using an optimized instrument design and analyzed the data using the three-compartment model (Fig. 4d). Interestingly, global fitting overestimated *k*_*a*_, and *k*_*d*_ (Fig. 4e,f) by ~1.2-fold at <10% MTL, which coincides with transient formation of the boundary layer at <3 *ms*. The error in *k*_*t*_ (Fig. 4g) followed an almost identical dependence on %MTL but was underestimated by as much as 2.5-fold. Interestingly, the error in *k*_*a*_, *k*_*d*_ and *k*_*t*_ and associated χ^2^ (Fig. 4h) were successfully negated over the full MTL range tested by simply pre-calculating *k*_*t*_, which is well proven in SPR-based calibration-free concentration measurement^29,30^.

In summary a temporal rank order where *t*_*grad*_ >τ> *t*_*wash*_ *and t*_*grad*_ >τ_ss_> *t*_*wash*_ is required where flow rate, and microchannel volume are chosen to achieve appropriate parameter ranges. As mentioned earlier, the analyte concentration in the flow cell can be assumed to follow a quasi-steady-state concentration when *t*_*grad*_≫ *t*_*wash*_. Essentially, the gradient should evolve gradually allowing occupancy to progress relatively slowly. Indeed the maximum change in occupancy observed in this simulation over the time period *t*_*wash*_ was <1% assuming *c*_3_ = *K*_*D*_. Taken together the measurement of transient kinetic processes requires avoiding abrupt changes in concentration and associated target occupancy thereby maintaining the quasi-steady state boundary layer assumption. In summary, we have developed a label-free biosensing approach to measure transient kinetics reporting an experimental proof-of-principle showing mechanistic characterization through transition state theory. These data and the numerical simulation studies establish the technical feasibility of the approach and support further development towards higher throughput applications in life science. It should also be noted that transient binding requires <1 s of sample exposure and may therefore be well suited to applications in high throughput screening.

## Methods

### General experimental

Assays were conducted using a Biacore S200 (GE Healthcare Bio-Sciences AB, SE-751 84, Uppsala, Sweden). All reagent coupling kits and sensors were from GE Healthcare. All reagents were from Sigma-Aldrich (3300 S. Second St., St. Louis, MO 63118, USA) unless otherwise stated. The MBB-tagged target were expressed recombinantly and purified in-house using standard protocols. All experiments were performed using a buffer containing 50 mM 4-(2-hydroxyethyl)-1-piperazineethanesulfonic acid, (HEPES), pH 7.5, containing 0.15 M sodium chloride and 0.2 mM tris(2-carboxyethyl)phosphine (TCEP). The 3,879 Da peptide was synthesized by GenScript (860 Centennial Ave, Piscataway, NJ 08854, USA) and purity was >95% by HPLC.

### Injection-binding process

A series S CM5 chip was employed and the analysis temperature was set to 20 °C. A series of dextran molecular weight standards were prepared from 1–10 mg/ml in standard buffer. Sucrose (*M*_*r*_ = 342 Da) was included as a low molecule *M*_*r*_ standard. Each standard was injected at 100 μL/min for 2 s in duplicate and each curve was normalized with respect to the maximum response for data analysis. Here we neglect double referencing and analyze bulk RI response curves without a binding component in order to characterize the quality of transient analyte dispersions.

### Experimental characterization in a moderate transient regime

A series S CM5 chip was employed and the analysis temperature was set to 5 °C. A MBP-tagged target (Mr ~52.3 kDa) was coupled according to manufacturers recommendations with the following changes. Seven serial-doubling dilutions of the 3,879 Da peptide (synthesized by Genescript, encompasses an SH3 recognition region) were prepared from 100 μM in standard buffer. Each sample was injected at 100 μL/min for 2 s in duplicate along with blank buffer injections for referencing thereby generating a curve set containing 14 binding curves. Each curve set was referenced against an averaged blank curve and fit to a three-compartment model using Biaevaluation 3.0 (GE Healthcare Bio-Science AB). This was repeated for at analysis temperatures of 10 °C, 15 °C, 20 °C, 25 °C, 30 °C and 35 °C.

### Experimental characterization- data analysis

The analysis of transient kinetic curve sets was essentially identical to conventional methods other than the inclusion of a dispersion term. The Biacore S200 supports 40 Hz data acquisition rates. The sensing regions of the Biacore S200 are in series causing a time delay to exist upon arrival of sample at the sensing region relative to its reference that overlaps entirely with the rise/fall regions needed for transient kinetic analysis. Single reference against a blank, as opposed to standard double referencing, prevented interference and the response curve contained both a bulk RI response and a binding response. The analytes bulk RI component is a good approximation of the analytes dispersion profile and a three-compartment model may be fit to this two-component response curve set by adding a bulk RI analyte dispersion defined by equation (S2). When fitting, we first determined both *K*_*D*_ and *R*_*max*_ from fitting a conventional steady-state model after double referencing. We fit a three-compartment model, holding *R*_*max*_ constant and global constraint of kinetic parameters. The slope coefficients (b, d) of equation (S2) were also constrained to global values while time coefficients (a, c) were fit locally.

### Experimental characterization- thermodynamic analysis

The temperature dependence of the kinetic and affinity constants obtained from fitting the three compartment model was analyzed by Eyring and Van’t Hoff analysis, respectively. The linear form of the Van’t Hoff equation is *ln*(*K*_*D*_) = *ΔH°/*(*RT*)−*ΔS°/R*, where *ΔH°* and ΔS° are the standard enthalpy and entropy of binding, respectively. *T* is the absolute temperature and *R* is the universal gas constant, 1.987 cal/ (mol K). A linearization plot of *Ln*(*Kd*) versus *1/T* was plotted and fit to the Van’t Hoff equation giving a slope *ΔH°/R* and an intercept -ΔS°/R. The linear form of the Eyring equation for the association process is given by *Ln*(*k*_*a*_*h/k*_*B*_*T*) = *−ΔH*^*ǂ*^*/RT* + *ΔS*^*ǂ*^*/R* where *ΔH*^*ǂ*^ and *ΔS*^*ǂ*^ are the changes in transition state enthalpy and entropy, respectively, associated with the formation of the affinity complex, *h* is Planks constant = 6.63 × 10^−34^ J s and *k*_*B*_ is Boltzmann constants = 1.38 × 10^−23^ J K^−1^, respectively. Substituting *k*_*d*_ for *k*_*a*_ gives the transition state analysis for complex dissociation. A linearization plot of *Ln*(*k*_*a*_*h/k*_*B*_*T*) versus *1/T* returns -*ΔH*^*ǂ*^*/R* and ΔS^*ǂ*^*/R* from the slope and intercept, respectively. The free energy was calculated from *ΔG* = *ΔH -TΔS* and was plotted as a function of the reaction coordinate^31^. The associated standard error bars are the SE of the fit associated with each parameter from the linearization plots. The analysis was performed using Graphpad Prism version 6 (GraphPad Software, Inc. 7825 Fay Avenue, Suite 230, La Jolla, CA, 92037, USA).

### Modeling

For details on modeling dispersion gradients, influence of mass transport limitation, general data analysis and statistics, correlation analysis and numerical simulations please refer to supplementary information that accompanies this paper.

## Supplementary information

Supplementary information

## References

[1] Van Regenmortel MH (2001). Analysing structure-function relationships with biosensors. Cell. Mol. Life Sci..

[2] Fierke CA, Hammes GG (1995). Transient kinetic approaches to enzyme mechanisms. Methods Enzymol..

[3] Perkinsm JR, Diboun I, Dessaillym BH, Lees JG, Orengo C (2010). Transient protein-protein interactions: structural, functional, and network properties. Structure.

[4] Albery WJ, Knowles JR (1976). Evolution of enzyme function and the development of catalytic efficiency. Biochemistry.

[5] Sweetlove LJ, Fernie AR (2018). The role of dynamic enzyme assemblies and substrate channelling in metabolic regulation. Nat. Commun..

[6] Kastritis PL, Bonvin AM (2013). On the binding affinity of macromolecular interactions: daring to ask why proteins interact. J. R. Soc. Interface.

[7] Schreiber G, Haran G, Zhou HX (2009). Fundamental aspects of protein-protein association kinetics. Chem. Rev..

[8] Renaud JP (2016). Biophysics in drug discovery: impact, challenges and opportunities. Nat. Rev. Drug Discov..

[9] Schilder J, Ubbink M (2013). Formation of transient protein complexes. Curr. Opin. in Struct. Biol..

[10] Burton RL, Hanes JW, Grant GA (2008). A stopped flow transient kinetic analysis of substrate binding and catalysis in Escherichia coli D-3-phosphoglycerate dehydrogenase. J. Bio. Chem..

[11] Sebastián M, Serrano A, Velázquez-Campoy A, Medina M (2017). Kinetics and thermodynamics of the protein-ligand interactions in the riboflavin kinase activity of the FAD synthetase from Corynebacterium ammoniagenes. Sci. Rep..

[12] Chi NC (2010). Deciphering the kinetic binding mechanism of dimeric ligands using a potent plasma-stable dimeric inhibitor of postsynaptic density protein-95 as an example. J. Bio. Chem..

[13] Myszka DG (2003). The ABRF-MIRG'02 study assembly state, thermodynamic, and kinetic analysis of an enzyme/inhibitor interaction. J. Biomol. Tech..

[14] Rich RL (2009). A global benchmark study using affinity-based biosensors. Anal. Biochem..

[15] Papalia GA (2006). Comparative analysis of 10 small molecules binding to carbonic anhydrase II by different investigators using Biacore technology. Anal. Biochem..

[16] Amaral M (2017). Protein conformational flexibility modulates kinetics and thermodynamics of drug binding. Nat. Commun..

[17] ResearchGate – SPR-Science topic, https://www.researchgate.net/topic/SPR/publications, access date Nov. 7th 2019.

[18] Copeland RA (2016). The drug-target residence time model: a 10-year retrospective. Nat. Rev. Drug Discov..

[19] Sykes DA (2017). Extrapyramidal side effects of antipsychotics are linked to their association kinetics at dopamine D2 receptors. Nat. Commun..

[20] McGovern, S. L., Caselli, E., Grigorieff, N. & Shoichet, B. K. A. Common mechanism underlying promiscuous inhibitors from virtual and highthroughput screening. *J. Med. Chem*. **45**(8), 1712–1722 (2002). 10.1021/jm010533y 11931626

[21] Sink R, Gobec S, Pečar S, Zega A (2010). False positives in the early stages of drug discovery. Curr. Med. Chem..

[22] Torosyan H, Shoichet BK (2019). Protein stability effects in aggregate-based enzyme inhibition. J. Med. Chem..

[23] Sjölander S, Urbaniczky C (1991). Integrated fluid handling system for biomolecular interaction analysis. Anal. Chem..

[24] Quinn JG (2012). Modeling of Taylor dispersion injections: determining kinetic/affinity interaction constants and diffusion coefficients in label free biosensing. Anal. Biochem..

[25] Quinn JG (2012). Evaluation of Taylor dispersion injections: determining kinetic/affinity interaction constants and diffusion coefficients in label free biosensing. Anal. Biochem..

[26] Squires TM, Messinger RJ, Manalis SR (2008). Making it stick: convection, reaction and diffusion in surface-based biosensors. Nat. Biotechnol..

[27] Hansen R, Bruus H, Callisen TH, Hassager O (2012). Transient convection, diffusion, and adsorption in surface-based biosensors. Langmuir.

[28] Myszka DG, He X, Dembo M, Morton TA, Goldstein B (1998). Extending the range of rate constants available for BIACORE: interpreting mass transport influenced binding data. Biophys. J..

[29] Christensen LLH (1997). Theoretical analysis of protein concentration determination using biosensor technology under conditions of partial mass transport limitation. Anal. Biochem..

[30] Pol E (2016). Evaluation of calibration free concentration analysis provided by Biacore™ systems. Anal. Biochem..

[31] de Mol NJ, Dekker FJ, Broutin I, Fischer MJE, Liskamp RMJ (2005). Surface plasmon resonance thermodynamic and kinetic analysis as a strategic tool in drug design. Distinct ways for phosphopeptides to plug into Src- and Grb2 SH2 domains. J. Med. Chem..

