# Supplementary material for: Unlocking latent kinetic information from label-free binding
Source: Sci Rep. 2019 Dec 5;9:18389. doi: 10.1038/s41598-019-54485-4 (PMC6895076; doi:10.1038/s41598-019-54485-4)
Supplement: Supplementary file 1 — Supplementary information [file 41598_2019_54485_MOESM1_ESM.pdf]

Supplementary Information

Unlocking latent kinetic information from label-free binding

John G. Quinn*[1] Micah Steffek[1], John M. Bruning[1], Alexandra Frommlet[1], and Melinda M. Mulvihill[1]

[1]Biophysical group, Biochemical and Cellular Pharmacology, Genentech, Inc., 1 DNA Way, South San Francisco, CA 94080

Correspondence and requests for materials should be addressed to J.G.Q

(email: quinnj6@gene.com)

Table of contents

Supplementary methods

Title	page number
Modeling dispersion gradients	3
Influence of MTL	3
General data analysis and statistics	3-4
Correlation analysis	4
Numerical simulations	4-6
Table S1	6
Supplementary methods references	6

Supplementary Methods

Modeling dispersion gradients. Analyte dispersion gradients form sigmoidal rise/fall profiles and may be modeled using an ODE, or an analytical expression[1] such as;

$$\frac{c_2}{c_1} = 0.5 - 0.5Erf\,\frac{1-\theta}{2\left(k/_{ul}\theta\right)^{0.5}} \tag{S1}$$

where c_2 = analyte concentration in flow cell (mol m^{-3}), c_1 = concentration of analyte injected (mol m^{-3}), Erf(z) = Gauss error function, where its complementary form is Erfc(z) and applies to a falling gradient, k = effective dispersion coefficient, θ = dimensionless time = t/τ_r, τ_r = mean analyte residence time (s) = L/u, L = length of microchannel (m) , u = average velocity of fluid (m s^{-1}). Equation (S1) is a biophysical expression relating the relevant physicochemical parameters and accommodates model fitting over a range of experimental conditions. Here we assume constant experimental conditions and avoid the computationally intensive Erf(z) of equation (S1) by using the following logistic equation to model dispersion,

$$\frac{c_2}{c_1} = \frac{\left(\frac{t}{a}\right)^b - \left(\frac{t}{c}\right)^d}{\left(\left(\frac{t}{c}\right)^d + 1\right)\left(1 + \left(\frac{t}{a}\right)^b\right)} \tag{S2}$$

where a, b, c, d are gradient fit coefficients. The fit coefficients may be pre-calculated by fitting equation (S2) to bulk refractive index (RI) dispersions but may also be estimated during fitting of the three-compartment model to binding curves. The time for the analyte dispersion to rise by 95% is $t_{grad} = a19^{1/b}$ *and* for the fall is $t_{grad} = c19^d$. From a practical perspective t_{grad} is readily controlled by flow rate selection and by employing a range of injection loops geometries. In the limit of $t_{grad} \ll \tau$ the uniform analyte concentration assumption holds such that the three-compartment model effectively reduces to a two-compartment model. When $t_{grad} \gg \tau$ then occupancy develops slowly relative to τ allowing systematic errors, such as baseline drift, to impact the data to a greater degree but this remains negligible for transient dispersion injections because $t_{grad} < 1s$.

Influence of mass transport limitation. When the surface reaction flux coefficient $k_r = k_a\,(R_{max}-R)$ is in the same order as k_t, or higher, then mass transport limitation $MTL = k_r/(k_r+k_t)$ will be significant and a compartment model that includes k_t is then required for reliable kinetic analysis.

$$k_t = 1.282\ 10^9 M_R \left(\frac{u_C D^2}{h^2 l}\right)^{\frac{1}{3}} \tag{S3}$$

where u_c is the maximum velocity through the center of the microchannel, D is the analyte diffusion coefficient and can be estimated from the Einstein-Stokes-Sutherland equation[2], h is the height of the flow cell and l is the length of the sensing region. Additional target-coated areas upstream are undesirable as this increases %MTL for a given R_{max} but when present this additional analyte binding sink requires an additional area correction factor.

Data analysis and statistics. Binding curves can be fit with a mechanistic interaction model by numerical integration of a coupled set of ordinary differential equations (ODE) combined with nonlinear squares curve fitting. Microsoft Excel and Biaevaluation (GE Healthcare Bio-Sciences AB) were employed for data processing. The computer generated image of the full injection-binding process shown in Fig. 1a was

generated in Comsol. Model fitting to binding curve sets was performed using Biaevaluation (GE Healthcare Bio-Sciences AB) and all images of fitted binding curve sets were taken directly from Biaevaluation. Graphpad Prism version 6 (GraphPad Software, Inc. 7825 Fay Avenue, Suite 230, La Jolla, CA, 92037, USA) was employed to plot Fig. 1b and Fig. 1d, parameter estimation error in Fig. 2 and Fig. 4 and for fitting thermodynamic models in Fig. 3b and presenting the transition state reaction plot in Fig. 3c. Microsoft excel was used to plot the evolution of the analyte concentration profile across the height of the flow cell at two flow rates (Fig. 4a). Curve fitting programs enable fitting of binding interaction data to interaction models by nonlinear regression, and the associated statistical methods to confirm goodness of fit and confidence in parameter estimates are well established[3]. Statistical parameters such as the standard error of the fit (SE) associated with a given parameter returned in the fit were used to report confidence in parameter estimates. The SE is a measure of the information content of the data and specifies the degree to which the curves define the parameter value from the fit. Values < 5% indicate high confidence and values >10% indicate that the parameter is poorly defined. The goodness of fit between a model curve and an experimental curve is described by χ^2 when the number of data points is high and by a regression coefficient R^2 when the number of values is low. χ^2 is the square of the averaged residual response difference and approaches the baseline noise for the best fits. Typically high quality fits will produce χ^2 values < 5%. Occasionally χ^2 may be within acceptable limits but the fit may remain questionable if residuals are not distributed randomly. A large systematic residual deviation can indicate inappropriate model fitting as observed in Fig. 2b.

Curves generated by numerical simulation follow deterministic algorithms and reproduce without error and therefore do not require replicates. However, simulation over a sufficiently wide parameter range is important in evaluating simpler models specified as ODEs and we choose ranges that were representative of practical experimental ranges. For a well-optimized assay, label-free binding technology can reproduce binding curves to a very high accuracy as observed in Fig. 3a. Each curve set contains duplicate curves at 6 concentrations. Duplicates are not averaged into an average curve but instead the model is fit simultaneously to all curves in the set producing 12 simulated curves that when well fit superimpose onto the experimental curves. All interaction parameters are constrained to a single global value over the entire set providing a robust test of the model. The resulting parameter estimates therefore represent the sum of multiple replicated measurements varied in concentration at a given temperature. Biaevaluation 3.0 was used to fit interaction models by writing a custom model using the general model fit editor in the form of the coupled ODE for a two-compartment model as given in Fig. 1c. and using equation (S2) to model dispersion and equation (S3) to pre-calculate k_t.

Correlation analysis. The correlation of model parameters was determined using Graphpad Prism by simulating a set of model curves using the three-compartment model where k_a = 1 x 10^6 M^{-1} s^{-1}, k_d = 10 s^{-1}, R_{max} = 60 RU and k_t = 1.15 x 10^8 m/s. The covariance matrix was automatically calculated by the program and was plotted as correlation coefficients in figure 2i.

Numerical simulations. This complex mathematical discipline requires specialists working with dimensionless numbers in order to collapse the near infinite possibilities of parameter space into the simplest governing theory applicable to a given regime of operation[4]. However, we confine our simulation studies to model specific experimental conditions rendering the added level of abstraction imposed by dimensionless analysis unnecessary. We employ terminology that is familiar to the drug discovery community in order to aid understanding. Comsol multiphysics 5.1 (COMSOL AB, Tegnérgatan 23, SE-111 40, Stockholm, Sweden) was used to perform all numerical simulations. A computational model replicating the biosensor system given in Fig. 1a was created. Typical microfluidic channels employed in biosensors have high aspect ratios where side walls are far apart relative to the top/bottom walls allowing microchannel width to be neglected thereby reducing the model to a cross-section through the

microchannel. The microchannel has height h = 50 μM and length l = 21 mm with a short flow cell at the exit region. A mesh composed of > 200 k elements was applied with exponentially closer spacing at the wall boundaries and the number of elements was increased until no detectable change was observed in the simulation output. The serpentine microchannel was included in Fig. 1 and Fig. 2 in order to generate a dispersion profile thereafter dispersion was defined using an analytical dispersion for all other data sets. Equation (S2) was used because the transient kinetics (τ = 10 ms) being simulated demanded a very high mesh density to ensure that the simulation was stable over all parameter ranges evaluated and resulted in impractically long computational times when applied over the entire serpentine microfluidic channel. The incompressible form of the Navier-Stokes equation was used to solve the two-dimensional velocity profile through the channel, assuming steady-state, a constant flow rate and atmospheric pressure. The velocity at the walls $u_{wall} = 0$, the inlet velocity $u_{inlet} = 0.1$ m s^{-1}, then solving for the velocity vector field over the full domain.

$$\nabla . u = 0 \tag{S4}$$

$$\rho u \cdot \nabla u = -\nabla p + \mu \nabla^2 u \tag{S5}$$

where ρ is the density, p is the pressure and μ is the dynamic viscosity.

The flow velocity vector field was coupled to the steady-state advection/diffusion equation for a dilute species to solve for the analyte concentration field in the bulk flow.

$$\nabla . (-\mathrm{D}\nabla \mathrm{c}) + u . \Delta c = R \tag{S6}$$

Here D is the diffusion coefficient, c is analyte concentration and R is a reaction term.
Initially the analyte concentration in the microchannel c = 0. At the inlet the initial analyte concentration profile along the microchannel height was defined by multiplying the concentration by a suitable scaling function (e.g. rectangular, sigmoidal, step, wave function). Target was assumed to be bound at the wall of the flow cell adjacent to the microchannel exit and required a surface reaction flux at that wall. The associated surface reactions were modeled by a *surface molar flux*, $N_{t,i}$ according to

$$N_t = -D_{si} \nabla_t c_{si} \tag{S7}$$

where $D_{s,i}$ is the surface diffusion coefficient for species *i at concentration* $c_{s,i}$. The governing equation for surface reaction is

$$\nabla_t . (-\mathrm{D}_s \nabla_t \mathrm{c}_s) = R_s \tag{S8}$$

where R_s is the surface flux balance (mol m^{-2}) with $D_s = 0$. The reversible binding reaction at the surface was given by a mechanistic 1:1 pseudo-first order interaction model identical in form to equation (1) given in terms of biosensor response but is presented here in terms of species concentrations where c_{max} is the total concentration of target bound to the sensing surface, c_s is the concentration of affinity complex at any given time.

$$\frac{\partial c_s}{\partial t} = k_a * c * (c_{max} - c_s) - k_d * c_s \tag{S9}$$

The binding flux of analyte to the target-coated sensing surface was balanced by a coupled flux loss from the bulk liquid. The time-dependent change in analyte accumulation was found from a surface flux balance at the sensing surface where the simulation was performed in time-stepping mode. The accumulation of affinity complex was expressed in terms of an equivalent biosensor response $R_{(t)} = c_{s(t)}.M_r.10^6$. Parameter values employed in numerical simulations are given in table S1.

Figure ID	k_a (x 10^6, M^{-1} s^{-1})	k_d (M^{-1} s^{-1})	R_{max} (x 10^{-9} mol m^{-3})	Coefficients a,b,c,d	u (m s^{-1})
Fig.1a	1	5	5000	-	0.01
Fig.2b, c	1	5	1	-	0.16
Fig.2d	1	10	30,60,120,240,480	-	0.10
Fig.2e	10	20	10, 100, 1000	0.50,10,1.5,17.8	0.10
Fig.2i	0.1	2	10	0.20,3.0,1.8,70.0	0.16
Fig. 4a	1	100	2.88	0.0026, 2.1, 0.0526, 15	0.1, 1.0
Fig. 4b	10,000	10,000	2.9, 4.3, 6.5, 9.7, 14.6, 21.9, 32.8, 49.3, 73.9, 110.9, 166.3	0.0026, 2.1, 0.0526, 15	1
Fig. 4d	10	100	2.9, 4.3, 6.5, 9.7, 14.6, 21.9, 32.8, 49.3, 73.9, 110.9, 166.3	0.0026, 2.1, 0.0526, 15	1

	Microchannel	Input Function	Analyte Concentration (μM)	D (x 10^{-10}, m^2 s^{-1})	Flow Cell (μm)
Fig.1a	Serpintine	Square	50,25,12.5,6.25,3.125,1.56	4.0	300 x 50
Fig.2b, c	Serpintine	Square	50,25,12.5,6.25,3.125,1.56	4.06	100 x 50
Fig.2d	Serpintine	Square	50,25,12.5,6.25,3.125,1.56	4.06	100 x 50
Fig.2e	Simple (1000 x 50)	Eq.S2	25	4.0	200 x 50
Fig.2i	Simple (1000 x 50)	Eq.S2	100, 50,25,12.5,6.25,3.125	4.0	200 x 50
Fig. 4a	Simple (300 x 50)	Eq.S2	10	3.0	100 x 50
Fig. 4b	Simple (300 x 50)	Eq.S2	50,25,12.5,6.25,3.125,1.56	4.08	100 x 50
Fig. 4d	Simple (300 x 50)	Eq.S2	50,25,12.5,6.25,3.125,1.56	4.08	100 x 50

Table S1. Parameter values employed in numerical simulations.

Supplementary methods references

1. Taylor, G. Dispersion of soluble matter in solvent flowing slowly through a tube. *Proc. R. Soc. London, A* **219,** 186–203 (1953).

2. Walters, R., Graham, J. F., Moore, R. M. & Anderson, D.J. Protein diffusion coefficient measurements by laminar flow analysis: method and applications. *Anal. Biochem.* **140,** 190-195 (1994).

3. Motulsky, H. J. & Christopoulos, A. Fitting models to biological data using linear and non-Linear regression. A practical guide to curve fitting. (Graphpad Software, Inc, San Diego, 2003).

4. Gervais, T. & Jensen, K.F. Mass transport and surface reactions in microfluidic systems. *Chem. Eng. Sci.* **61,** 1102–1121 (2006).
